# Primary ovarian insufficiency in classic galactosemia: role of FSH dysfunction and timing of the lesion

**DOI:** 10.1007/s10545-012-9497-7

**Published:** 2012-06-23

**Authors:** Cynthia S. Gubbels, Jolande A. Land, Johannes L. H. Evers, Jörgen Bierau, Paul P. C. A. Menheere, Simon G. F. Robben, M. Estela Rubio-Gozalbo

**Affiliations:** 1 Departments of Pediatrics and Clinical Genetics, Maastricht University Medical Center, Maastricht, The Netherlands; 2 Department of Obstetrics and Gynaecology, University Medical Center Groningen, Groningen, The Netherlands; 3 Department of Obstetrics and Gynaecology, Maastricht University Medical Center, Maastricht, The Netherlands; 4 Genetic Metabolic Disease Laboratory, Maastricht University Medical Center, Maastricht, The Netherlands; 5 Department of Clinical Chemistry, Maastricht University Medical Center, Maastricht, The Netherlands; 6 Department of Radiology, Maastricht University Medical Center, Maastricht, The Netherlands; 7 Department of Pediatrics and Genetic Metabolic Disease Laboratory, Maastricht University Medical Center, Maastricht, The Netherlands; 8 Department of Pediatrics, Maastricht University Medical center, P.O. Box 5800, NL-6202 AZ Maastricht, The Netherlands

## Abstract

FSH inactivity due to secondary hypoglycosylation has been suggested as a potential mechanism for primary ovarian insufficiency in classic galactosemia. To investigate the role of FSH and to gain insight in the timing of the damage, ovarian stimulation tests were performed and data on ovarian imaging collected. Fifteen patients with primary ovarian insufficiency underwent ovarian stimulation with gonadotropins. Only one patient showed a normal increase in estradiol level, all the others had a low or no estradiol response. Anti-Müllerian hormone measurement in all girls and women showed levels below the detection limit of 0.10 μg/l. Ovarian volumes were evaluated by MRI in 14 patients and compared to age matched controls, prepubertal controls and postmenopausal controls. The ovarian volumes of the galactosemic girls were smaller than those of the age matched controls (p = 0.001) and the prepubertal ovaries (p = 0.008), and did not differ significantly from postmenopausal ovarian volumes (p = 0.161). In conclusion we found no evidence that FSH inactivity plays a role in primary ovarian insufficiency in classic galactosemia. Moreover, ovarian imaging results point to an early onset of ovarian failure in this disease.

## Introduction

Primary ovarian insufficiency (POI) is frequent in classic galactosemia (Holton et al 2001; Kaufman et al 1981). According to female patients and their parents, this complication represents the greatest psychological burden for women with this disorder (Bosch et al 2004). Most patients experience hypergonadotropic hypogonadism, with subsequent absent or delayed pubertal development, amenorrhea/oligomenorrhea and decreased fertility. Usually they are treated with exogenous estrogens to prevent complications of hypoestrogenism.

The etiology underlying POI in classic galactosemia is not yet fully understood, and several mechanisms have been postulated including direct toxicity of metabolites, altered gene expression or aberrant function of hormones and or receptors due to glycosylation abnormalities (Rubio-Gozalbo et al 2009). It is also conceivable that not one, but the different mechanisms acting in unison are responsible for this clinical picture. Galactose or galactose metabolites could induce damage, either through a direct or an indirect toxic effect on the ovary, with chronically increased apoptosis leading to ovarian atrophy (Kaufman et al 1986). The galactose burden in this disease also seems to alter gene expression (Coman et al 2010; Lai et al 2008). In addition, classic galactosemia leads secondarily to glycosylation abnormalities of complex molecules, possibly through inhibition of galactosyltransferases by Gal-1-P. Glycosylation is often crucial for an adequate function of these complex molecules. (Charlwood et al 1998; Dobbie et al 1990; Haberland et al 1971; Jaeken et al 1992; Ornstein et al 1992; Petry et al 1991; Staubach et al 2011; Stibler et al 1997; Sturiale et al 2005).

FSH, a glycoprotein, is highly dependent on glycosylation (Combarnous 1992) for its function, and theoretically, an altered FSH function due to hypoglycosylation in this disease, could lead to primary ovarian insufficency. Studies on the glycosylation pattern of FSH in women with classic galactosemia and POI, show different results. Prestoz et al (1997) found in their study hypoglycosylated FSH isoforms in these patients, whereas our group found a normal FSH isoform pattern (Gubbels et al 2011). If FSH inactivity is a key mechanism causing POI in this disease, one would expect a rise in estradiol when exogenous, active FSH is given.

There is also uncertainty about when exactly the damage occurs. Observations in animal models suggest a prenatal onset of the lesion. Rats exposed to galactose prenatally had a decreased number of oocytes (Chen et al 1981), and rats with pre- and postnatal galactose exposure had overall ovarian dysfunction (Bandyopadhyay et al 2003). In humans however, normal ovarian histology has been observed in two neonates (Levy 1996; Levy et al 1984), as well as normal appearance of the ovaries in a girl at age seven (Kaufman et al 1981), whereas later in adolescence she had atrophic ovaries.

In this study, a group of adolescent and adult female classic galactosemia patients was evaluated using ovarian stimulation and ovarian imaging to address the following questions:

- Is there evidence for FSH dysfunction?
- When does the damage to the ovaries occur?

## Patients and methods

### Patients

A total of 24 female galactosemia patients, aged 6–43, were included. All studies were approved by our IRB. Patients were invited to participate through our outpatient clinic and by the Dutch Galactosemia Association from March 2007 – October 2009 and volunteered with informed consent. The inclusion criterion for these studies was confirmed classic galactosemia diagnosis (GALT enzyme activity measurement and/or GALT gene mutational analysis). The patients who participated in the stimulation protocols had all POI defined as amenorrhea for at least four months before the age of 40 and confirmed by two separate measurements of FSH in the postmenopausal range with at least 1 month between measurements (Nelson et al 2009). Patient characteristics are summarized in Table 1.

**Table 1:** Patient characteristics

Nr	Age (years)	Mutation	GALT-activity (nmol/mmol Hb/hr (% of normal control))	Exposure to lactose (days)	Spontaneous menarche (age (years))	Age at POI diagnosis (years)	FSH (IU/l) at POF diagnosis	Participated in
1	19	Q188R/ Q188R		8	Yes (12)	14	65.6	Stimulation, MRI
2	29	Q188R/ Q188R		10	No	16	83.0	Stimulation
3^1^	24	Q188R/S135W	3.7 (0.7 %)	‘Neonatally’ diagnosed	Yes (16)	19	NA	Stimulation
4	22	Q188R/ Q188R	21.2 (3.7 %)	3	No	6	73.0	Stimulation
5	23	Q188R/ Q188R		7	No	1.5	30.0	Stimulation
6^2^	36	Q188R/ K285N	4.5 (0.8 %)	15	Yes (16)	27	89.0	Stimulation
7	21	Q188R/ Q188R	18.3 (3.1 %)	0	Yes (12)	12	34.0	Stimulation, MRI
8	21	Q188R/Q188R	0.4 (0.1 %)	11	Yes (13)	17	48.5	Stimulation
9	24	Q188R/ Q188R	5.3 (0.9 %)	30	Yes (12)	15	>50	Stimulation
10	23	L195P/ L195P	21.8 (3.7 %)	14	No	12	57.5	Stimulation
11	27	L195P/ X380C^3^	14.5 (2.5 %)	NA	No	12	NA	Stimulation
12	15	Q188R/ Q188R		NA	No	12	82.5	Stimulation, MRI
13	15	IVSC/ IVSC	6.2 (1.1 %)	6	No	11	43.7	Stimulation
14	16	Q188R/Q188R		6	No	2	46.8	Stimulation, MRI
15	19	Q188R/ Q188R	3.9 (0.7 %)	NA	No	Unknown	NA	Stimulation, MRI
16	18	Q188R/ Q188R		NA	Yes (15)	Unknown	NA	MRI
17	16	Q188R/Q188R		9	No	10	54.2	MRI
18	11	L195P/ K229N		6	No	10	67.6	MRI
19	11	Q188R/Q188R		9	-	-	-	MRI
20	9	Q188R/Q188R	1.0 (0.2 %)	NA	-	-	-	MRI
21	9	Q188R/Q188R		23	-	-	-	MRI
22	14	Q188R/ Q188R	4.7 (0.8 %)	0	No	Unknown	NA	MRI
23	9	Q188R/Q188R	1.3 (0.2 %)	0	Yes (12)	-	NA	MRI
24	14	Q188R/Q188R	1.6 (0.3 %)	0	No	-	NA	MRI

Nr = Number

GALT = Galactose-1-phosphate Uridyl Transferase

POF = Premature ovarian insufficiency

NA = not available

^1^ 3 pregnancies: 1 stillbirth, 2 healthy children

^2^ 4 pregnancies: 2 miscarriages, 2 healthy children

^3^ Bosch et al 2005

Control patients in three control groups (age matched, pre-pubertal and postmenopausal) for the MRI studies were sought anonymously from the database of MRI scans in our hospital. We ensured that the imaging used for control patients was comparable to the galactosemia ovary protocol. The majority of the control patients underwent an abdominal MRI for Crohn’s disease. Patients with known malformations of the reproductive tract or diseases known to affect the reproductive system were excluded. For every galactosemia patient, the two available controls that were closest in age to the patient at the time of the MRI were selected as age-matched controls. In all but one case, the age of the matched controls differed <1 year from the age of the patient. The only exception was a patient aged 8.9 years. In this case, as no other controls were available for her, the accepted matched controls were 7.0 years. The prepubertal control patients were 0.3 to 6.9 years old (mean 3.2 years), and their ovaries and uterus had no signs of pubertal development. All available suitable prepubertal patients were selected for this control group. In the Netherlands the average age at menopause is 51.5 + 3.9 years (Brand 1978). Therefore, the postmenopausal control patients were all >60 years old (mean 63.2 years; range 60.7-65.9 years). We selected the most recent suitable patients.

### Stimulation tests

Different stimulation tests were performed using a model wherein the insights gained in the previous test were applied to the following test.

1. EFORT

An exogenous FSH ovarian reserve test (EFORT) was performed in three patients with 300 IU recombinant FSH, Gonal-F (Serono Europe Ltd., Houten, The Netherlands). In the women who used oral contraceptives (OC) or hormone replacement therapy (HRT) prior to the test, their medication was withdrawn for seven days to eliminate external hormonal influences. Patients undergoing this test had subsequently a high endogenous FSH. Before the administration of the FSH and 24 hours after, 17β-estradiol was measured. An increase in 17β-estradiol >0.11 nmol/l is considered a positive response (Fanchin et al 1994).

2 Three day stimulation with FSH and LH

Nine patients were stimulated with a three day protocol that combined daily subcutaneous administration of FSH and LH (225 IU of Gonal-F and 150 IU of recombinant LH, Luveris (Serono Europe Ltd., Houten, The Netherlands)), or 225 IU of human Menopausal Gonadotropin (hMG), Menopur (Ferring B.V., Hoofddorp, The Netherlands)). Preceding the first injection, and 24 hours after the last one, 17β-estradiol was measured. To ensure that the patient’s own, possibly inactive, FSH could not block the FSH receptor (Combarnous 1992), all patients continuously used OC in the three weeks previous to and during the stimulation to inhibit their endogenous FSH production and ensure low levels of endogenous FSH.

3 Twenty day stimulation with FSH and LH

Three patients were stimulated with a 20 day protocol while using OC. During the first ten days, they received 150 IU of Menopur s.c. (kindly provided by Ferring BV) per day. If no response was seen, the study was continued with 225 IU Menopur s.c. per day during ten more days. 17β-estradiol was measured on days 1, 9, 11, 19 and 21. The rationale behind this longer stimulation period was that a 3-day stimulation might have been too short to see an estradiol response.

### Laboratory measurements

#### Hormonal values

Sera from the patients were collected following standard procedures. Commercially available immunofluorometric assays (IFMAs) were used for the determination of LH and FSH (PerkinElmer’s AutoDelfia, Turku, Finland). For 17-β-estradiol, we used a fluorometric immunoassay (FIA) (PerkinElmer’s AutoDelfia, Turku, Finland). AMH concentrations were quantitatively determined in a random sample (not during gonadotropin stimulation) using a commercially available assay from DSL (Webster, Texas, USA). The obtained laboratory values were compared to the control range used in Maastricht University Medical Center for female patients in the age group of our patients.

#### Statistical analyses

Patient characteristics, such as age at diagnosis/treatment, spontaneous menarche versus induced pubertal development, genotype and GALT enzyme activity were used to try to create a predictive model for estradiol response. SPSS version 16.0 was used for analysis.

### Ovarian imaging

#### MRI

Magnetic resonance imaging was performed with a 1.5 T system. Transverse, sagital and coronal T2-weighted 2 mm slices covered the area between the umbilicus and symphysis pubis. Additional T1-weighted transverse slices and T2-weighted transverse slices with fat-suppression were performed. Ovarian volume was calculated by using the formula of an ellipsoid (length x width x depth x π/6).

#### Statistical analysis

Distribution of the samples was evaluated by plotting the data. As the samples were not normally distributed, non-parametric statistical tests were used. The ovarian volumes of the galactosemic girls were compared to age matched controls by using the Wilcoxon signed ranks test. For this, the mean volume of the two ovaries of each patient was compared to the mean volume of the four ovaries of the control patients. The mean ovarian volume of the galactosemia patients was compared to the mean volume of the prepubertal and postmenopausal control groups using the Mann–Whitney test. For analysis, in the cases where the ovaries could not be detected, we used the detection limit of 0.2 × 0.2 × 0.2 × π/6 = 0.004 cc, because 2 mm was both the smallest visualized ovarian dimension, as well as the distance between two slides. SPSS version 16.0 was used for analysis.

## Results

### Stimulation tests

The results of the stimulation tests are summarized in Table 2. No patient responded to the EFORT, but three out of nine patients showed an increase in 17β-estradiol concentration after stimulation with the 3 day regime of FSH and LH and two of three showed a response after 21 day stimulation. Only one (3 day) patient, however, showed a normal response of >0.3 nmol/l. Spontaneous menarche versus induced pubertal development, genotype and GALT enzyme activity were not predictive of an estradiol response during the simulation tests.

**Table 2:** Stimulation tests

Patient	Age (years)	Δ17β-estradiol (nmol/l)		
		EFORT (no OC)	3 day FSH/LH protocol (during OC)	21 day FSH/LH protocol (during OC)
1	19	0.0	-	-
2	29	0,0	-	-
3	24	0,0	-	-
4	22	0.0	-	-
5	23	0.0	0.0	-
6	36	0.0	0.1*	-
7	21	0.0	0.0	-
8	21	0.0	0.0	-
9	24	0.0	0.0	0.0
10	23	-	-	0.1*
11	27	-	-	0.2*
12	15	-	0.0	-
13	15	-	0.1*	-
14	16	-	0.0	-
15	19	-	0.3*	-

EFORT = Exogenous FSH ovarian reserve test

OC = Oral contraceptives

- = not performed

* Statistically significant response

### Ovarian imaging

Compared to age-matched controls, the ovaries of galactosemic girls and women were significantly smaller (*p* = 0.001). The ovaries of the galactosemia patients were also significantly smaller (*p* = 0.008) than those of control prepubertal girls but did not differ significantly (*p* = 0.161) from postmenopausal controls. The median ovarian volume was <0.004 cm^3^ (undetectable) (range <0.004-3.4 cm^3^) in galactosemia patients, 0.9 cm^3^ (range <0.004-3.7 cm^3^) in prepubertal girls and <0.004 cm^3^ (range <0.004-1.1 cm^3^) in the postmenopausal control group. The age matched and prepubertal control patients showed evidence of ovarian activity (multiple cysts) whereas in the galactosemic patients and in postmenopausal controls, evidence of activity was seen only rarely.

### Hormone assays

All patients had AMH levels below the detection limit of 0.10 μg/l . In the three patients that underwent 21 day stimulation, AMH was measured at the same time as 17β-estradiol, but no increases in AMH were seen. Spontaneous baseline FSH and LH levels in the non-prepubertal girls and women were in the postmenopausal range. FSH rose as expected during the stimulation tests, LH remained around the same level, as its half-life is shorter. For the 21 day stimulation, Menopur was used (hCG substitutes LH activity). All baseline 17β-estradiol levels were low for the patient’s age.

## Discussion

The response to FSH stimulation was evaluated in a group of adolescent and adult female classic galactosemia patients. If endogenous FSH dysfunction due to abnormal glycosylation is a key mechanism in this disease, the patients are expected to have a significant response to FSH stimulation analogous to patients with FSH beta subunit mutations (Matthews et al 1993; Rabin et al 1972). However, only one patient showed a significant response to FSH administration supporting the proposed mechanism that in the ovaries of classic galactosemia patients the numbers of follicles that can respond to exogenous gonadotropins is very low. These findings are in line with reports of normal bioactivity of endogenous FSH in galactosemia (Kaufman et al 1981; Sanders et al 2009). Interestingly, two patients (numbers 5 and 12) had a spontaneous menstruation for the first time within two months after the ovarian stimulation test, while they were still on their continuously dosed estrogen-only HRT. Another patient became pregnant two months after an EFORT (Gubbels et al 2009). This might be a coincidence and reflect sporadic ovulation or reflect that a follicle in a very early stage was stimulated to develop under FSH administration. The two month window is the needed time to progress from a secondary to an antral follicle (Gougeon 1986). Sporadic follicle maturation despite POI (Gubbels et al 2008) occurs and during the studies we witnessed this process. Where an MRI in patient 16 did not reveal detectable ovarian tissue, a transvaginal ultrasound performed five months later showed a clearly visible ovary with a cyst suggestive of a follicle of 2 cm diameter. This patient is currently 20 years old and spontaneously pregnant.

Regarding the question when the damage to the ovary occurs, imaging of the ovaries in all galactosemic girls and women revealed low mean ovarian volumes, similar to the ovaries of the postmenopausal women. The prepubertal ovaries of the controls were also significantly larger than the ovaries of the galactosemic group. These data are in agreement with ovarian damage occurring early in life. Demarcation of the time window in which ovarian damage occurs is very important in view of fertility maintenance strategies. Cryopreservation of ovarian tissue or vitrification of oocytes are current fertility preservation techniques increasingly applied in other diseases. The chances of fertility using these procedures depend on the ovarian reserve, and the use of these methods for this disease might only be a realistic option at a very early age and/or for selected candidates.

Within the limitations of the study because of the small and heterogeneous sample with a wide age range, we think this paper adds valuable information to the existing knowledge on POI in female classic galactosemia patients. We have not found evidence for FSH inactivity as the primary mechanism causing POI and damage to the ovary occurs early in life.

## References

[CR1] Bandyopadhyay S, Chakrabarti J, Banerjee S (2003). Prenatal exposure to high galactose adversely affects initial gonadal pool of germ cells in rats. Hum Reprod.

[CR2] Bosch AM, Grootenhuis MA, Bakker HD, Heijmans HS, Wijburg FA, Last BF (2004). Living with classical galactosemia: health-related quality of life consequences. Pediatrics.

[CR3] Bosch AM, IJlst L, Oostheim W (2005). Identification of novel mutations in classical galactosemia. Human Mutation.

[CR4] Brand PC (1978) Age at menopause (dissertation). Utrecht: Rijksuniversiteit Utrecht

[CR5] Charlwood J, Clayton P, Keir G, Mian N, Winchester B (1998). Defective galactosylation of serum transferrin in galactosemia. Glycobiology.

[CR6] Chen YT, Mattison DR, Feigenbaum L, Fukui H, Schulman JD (1981). Reduction in oocyte number following prenatal exposure to a diet high in galactose. Science.

[CR7] Coman DJ, Murray DW, Byrne JC (2010). Galactosemia, a single gene disorder with epigenetic consequences. Pediatr Res.

[CR8] Combarnous Y (1992). Molecular basis of the specificity of binding of glycoprotein hormones to their receptors. Endocr Rev.

[CR9] Dobbie JA, Holton JB, Clamp JR (1990). Defective galactosylation of proteins in cultured skin fibroblasts from galactosaemic patients. Ann Clin Biochem.

[CR10] Fanchin R, de Ziegler D, Olivennes F, Taieb J, Dzik A, Frydman R (1994). Endocrinology: exogenous follicle stimulating hormone ovarian reserve test (EFORT): a simple and reliable screening test for detecting 'poor responders' in in-vitro fertilization. Hum Reprod.

[CR11] Gougeon A (1986). Dynamics of follicular growth in the human: a model from preliminary results. Hum Reprod.

[CR12] Gubbels CS, Kuppens SM, Bakker JA (2009). Pregnancy in classic galactosemia despite undetectable anti-Mullerian hormone. Fertil Steril.

[CR13] Gubbels CS, Land JA, Rubio-Gozalbo ME (2008). Fertility and impact of pregnancies on the mother and child in classic galactosemia. Obstet Gynecol Surv.

[CR14] Gubbels CS, Thomas CM, Wodzig WK (2011). FSH isoform pattern in classic galactosemia. J Inherit Metab Dis.

[CR15] Haberland C, Perou M, Brunngraber EG, Hof H (1971). The neuropathology of galactosemia. A histopathological and biochemical study. J Neuropathol Exp Neurol.

[CR16] Holton JB, Walter JH, Tyfield LA (2001) Galactosaemia. In: Scriver CR, Beaudet AL, Sly WS, Valle D, Childs B, Kinzler KW, Vogelstein B (eds) The metabolic & molecular bases of inherited disease. 1553–1587

[CR17] Jaeken J, Kint J, Spaapen L (1992). Serum lysosomal enzyme abnormalities in galactosaemia. Lancet.

[CR18] Kaufman FR, Donnell GN, Roe TF, Kogut MD (1986). Gonadal function in patients with galactosaemia. J Inherit Metab Dis.

[CR19] Kaufman FR, Kogut MD, Donnell GN, Goebelsmann U, March C, Koch R (1981). Hypergonadotropic hypogonadism in female patients with galactosemia. N Engl J Med.

[CR20] Lai K, Tang M, Yin X, Klapper H, Wierenga K, Elsas L (2008). ARHI: a new target of galactose toxicity in classic galactosemia. Biosci Hypotheses.

[CR21] Levy HL (1996). Reproductive effects of maternal metabolic disorders: implications for pediatrics and obstetrics. Turk J Pediatr.

[CR22] Levy HL, Driscoll SG, Porensky RS, Wender DF (1984). Ovarian failure in galactosaemia. N Engl J Med.

[CR23] Matthews CH, Borgato S, Beck-Peccoz P (1993). Primary amenorrhoea and infertility due to a mutation in the beta-subunit of follicle-stimulating hormone. Nat Genet.

[CR24] Nelson SM, Yates RW, Lyall H et al (2009) Anti-Mullerian hormone-based approach to controlled ovarian stimulation for assisted conception. Hum Reprod 10.1093/humrep/den480 19136673

[CR25] Ornstein KS, McGuire EJ, Berry GT, Roth S, Segal S (1992). Abnormal galactosylation of complex carbohydrates in cultured fibroblasts from patients with galactose-1-phosphate uridyltransferase deficiency. Pediatr Res.

[CR26] Petry K, Greinix HT, Nudelman E (1991). Characterization of a novel biochemical abnormality in galactosemia: deficiency of glycolipids containing galactose or N-acetylgalactosamine and accumulation of precursors in brain and lymphocytes. Biochem Med Metab Biol.

[CR27] Prestoz LLC, Couto AS, Shin YS, Petry KG (1997). Altered follicle stimulating hormone isoforms in female galactosaemia patients. Eur J Pediatr.

[CR28] Rabin D, Spitz I, Bercovici B (1972). Isolated deficiency of follicle-stimulating hormone. Clinical and laboratory features. N Engl J Med.

[CR29] Rubio-Gozalbo ME, Gubbels CS, Bakker JA, Menheere PP, Wodzig WK, Land JA (2009). Gonadal function in male and female patients with classic galactosemia. Hum Reprod Update.

[CR30] Sanders RD, Spencer JB, Epstein MP (2009). Biomarkers of ovarian function in girls and women with classic galactosemia. Fertil Steril.

[CR31] Staubach S, Schadewaldt P, Wendel U, Nohroudi K, Hanisch F-G (2011) Differential glycomics of epithelial membrane glycoproteins from urinary exovesicles reveals shifts toward complex-type N-glycosylation in classical galactosemia. Journal of Proteome Research 10.1021/pr200711w 22087537

[CR32] Stibler H, von Döbeln U, Kristiansson B, Guthenberg C (1997). Carbohydrate-deficient transferrin in galactosaemia. Acta Paediatr.

[CR33] Sturiale L, Barone R, Fiumara A (2005). Hypoglycosylation with increased fucosylation and branching of serum transferrin N-glycans in untreated galactosemia. Glycobiology.

